# Genetic diversity and structure of Iberian Peninsula cowpeas compared to world-wide cowpea accessions using high density SNP markers

**DOI:** 10.1186/s12864-017-4295-0

**Published:** 2017-11-21

**Authors:** Márcia Carvalho, María Muñoz-Amatriaín, Isaura Castro, Teresa Lino-Neto, Manuela Matos, Marcos Egea-Cortines, Eduardo Rosa, Timothy Close, Valdemar Carnide

**Affiliations:** 1 Centre for Research and Technology of Agro-Environmental and Biological Sciences (CITAB), University of Trás-os-Montes and Alto Douro (UTAD), 5000-801 Vila Real, Portugal; 2 Department of Botany and Plant Sciences, University of California Riverside, Riverside, CA 92521-0124 USA; 3 Department of Genetics and Biotechnology, University of Trás-os-Montes and Alto Douro (UTAD), 5000-801 Vila Real, Portugal; 4 Biosystems & Integrative Sciences Institute (BioISI), Plant Functional Biology Center (CBFP), University of Minho, Campus de Gualtar, 4710-057 Braga, Portugal; 5 Biosystems & Integrative Sciences Institute (BioISI), Sciences Faculty, University of Lisbon, Campo Grande, 1749-016 Lisbon, Portugal; 6 Instituto de Biotecnología Vegetal, Universidad Politécnica de Cartagena, 30202 Cartagena, Spain

**Keywords:** *Vigna unguiculata*, Single nucleotide polymorphism, Genetic diversity and variation, Population structure

## Abstract

**Background:**

Cowpea (*Vigna unguiculata* L. Walp) is an important legume crop due to its high protein content, adaptation to heat and drought and capacity to fix nitrogen. Europe has a deficit of cowpea production. Knowledge of genetic diversity among cowpea landraces is important for the preservation of local varieties and is the basis to obtain improved varieties. The aims of this study were to explore diversity and the genetic structure of a set of Iberian Peninsula cowpea accessions in comparison to a worldwide collection and to infer possible dispersion routes of cultivated cowpea.

**Results:**

The Illumina Cowpea iSelect Consortium Array containing 51,128 SNPs was used to genotype 96 cowpea accessions including 43 landraces and cultivars from the Iberian Peninsula, and 53 landraces collected worldwide. Four subpopulations were identified. Most Iberian Peninsula accessions clustered together with those from other southern European and northern African countries. Only one accession belonged to another subpopulation, while two accessions were ‘admixed’. A lower genetic diversity level was found in the Iberian Peninsula accessions compared to worldwide cowpeas.

**Conclusions:**

The genetic analyses performed in this study brought some insights into worldwide genetic diversity and structure and possible dispersion routes of cultivated cowpea. Also, it provided an in-depth analysis of genetic diversity in Iberian Peninsula cowpeas that will help guide crossing strategies in breeding programs.

**Electronic supplementary material:**

The online version of this article (10.1186/s12864-017-4295-0) contains supplementary material, which is available to authorized users.

## Background

Cowpea (*Vigna unguiculata* L. Walp., 2n = 2× = 22) is a member of the Fabaceae family and one of the most important grain legumes growing in tropical and subtropical regions [1]. Grain-type cowpea, also known as common cowpea or African cowpea belongs to subspecies *unguiculata* while vegetable cowpea, commonly known as asparagus bean or ‘yardlong’ bean, belongs to subspecies *sesquipedalis* [2]. These two subspecies are differentiated mainly by their plant architecture, pod size and thickness, and end use [3, 4], but they both possess a high protein content [3, 5]. Other important characteristics of cowpea are the capacity to fix atmospheric nitrogen through symbiosis with root nodule bacteria [6], the ability to grow in low fertility soils [7], and the high tolerance to high temperatures and drought [8]. These attributes make cowpea a key crop in the context of global climate change and food security. In Southern Europe, namely the Iberian Peninsula, rainfall is projected to decrease while temperature is projected to increase [9].

Cowpea is native to Africa [10, 11] although the center of domestication is still uncertain. In the Neolithic period, cowpea was first introduced into India, which is now considered a secondary center of genetic diversity [12]. Some reports suggest that cowpea has been cultivated in Europe at least since the eighteenth century BC and possibly since prehistoric times [13, 14], while others suggest that it was introduced in Europe around 300 BC, where it still remains as a minor crop in the southern part of the continent. These two scenarios are not mutually exclusive. From Europe, more specifically from Spain, it has been speculated that cowpea was exported in the seventeenth century to the New World [15–17].

Assessment of the genetic diversity within a crop’s germplasm is fundamental for crop improvement and selection [1]. Moreover, the utilization of landraces is valuable as they can contain favorable alleles for many agronomic traits [18]. Until now, Iberian Peninsula cowpeas, including landraces, have not been genetically characterized, which is a prerequisite for their full exploitation in breeding. Recently, an iSelect BeadArray which assays 51,128 SNPs has been developed for cowpea and used to generate a consensus genetic map containing 37,372 SNPs and to assess genetic diversity within West African breeding materials [19], and to better understand the genetic basis underlying pod length variation [2].

Europe has a deficit of grain legumes, including cowpea. Imports into Europe were about 1.7 million tonnes worth 1.3 billion € in 2015 [20]. The recently developed Cowpea iSelect Consortium Array [19] provides an opportunity to use this tool to understand diversity in Iberian Peninsula cowpea germplasm and to apply this knowledge to breeding varieties producing higher and stable yields in the hotter, drier summers of Southern Europe. The main objectives of this study were to: (1) understand genetic diversity and structure in a set of Iberian Peninsula cultivated cowpea accessions in comparison to a worldwide collection of cowpea accessions; and (2) infer possible dispersion routes of cultivated cowpea, focusing on the contribution of the Iberian Peninsula cowpea germplasm.

## Methods

### Plant material

A total of 96 cowpea accessions from twenty-four countries were used in this study. They included 33 accessions from Portugal, 10 accessions from Spain (for a total of 43 accessions representing the diversity of Iberian Peninsula germplasm), and 53 accessions from genebanks at the National Institute for Agrarian and Veterinarian Research (INIAV, Portugal), the National Plant Genetic Resources Centre-National Institute for Agricultural and Food Technology Research (CRF-INIA, Spain), the Leibniz Institute of Plant Genetics and Crop Plant Research (IPK, Gatersleben, Germany), the Botanic Garden Meise (Belgium), the University of Perugia (Italy), and the Brazilian Agricultural Research Corporation (EMBRAPA, Brazil). These 53 accessions were chosen to represent worldwide cowpea diversity (Additional file 1). From these 96 accession, 86 belonged to ssp. *unguiculata*, while 10 were part of the ssp. *sesquipedalis*.

Leaves from three individual plants of each accession were collected. Total genomic DNA from each plant was extracted from 50 mg of well-developed trifoliate leaves (two-weeks-old) with the NucleoSpin® Plant II kit (Macherey-Nagel, Düren, Germany) using the Lysis Buffer 1 (based on the CTAB method) and the standard protocol according to the manufacturer’s instructions. DNA concentrations were measured using a NanoDrop 1000 (Invitrogen, California, USA). In order to verify DNA integrity, 2 μL of DNA were subjected to gel electrophoresis on 1.0% (*w*/*v*) agarose gel, stained with ethidium bromide. Equal amounts of the three DNA samples of each accession were bulked for genotyping to get a better estimation of diversity within each accession/bulk.

### SNP genotyping and data curation

The 96 accessions were genotyped with the Illumina Cowpea iSelect Consortium Array containing 51,128 SNPs [19] at the University of Southern California Molecular Genomics Core Facility (Los Angeles, CA, USA). SNPs included in this iSelect array were discovered in a panel of 37 phenotypically and genetically diverse accessions of cultivated cowpea from 12 countries in Africa, China and the USA, and included four accessions of ssp. s*esquipedalis* (Muñoz-Amatriaín et al. [19]). SNP calling was performed in GenomeStudio v.2011.1 software (Illumina Inc., San Diego, CA, USA) using the same cluster file as in Muñoz-Amatriaín et al. [19]. Quality control filters were applied to both SNPs and samples: first, SNPs with missing data and/or heterozygous calls in >20% accessions were eliminated; second, accessions with >20% missing SNP calls (which may be indicative of poor DNA quality) and/or >20% heterozygous calls were removed from further analysis. The 20% heterozygosity threshold was chosen based on outcrossing rates from 1 to 15% reported for cultivated cowpea [3, 21, 22]. In addition, SNPs were used to identify potentially identical individuals in the collection by performing pair-wise comparisons.

### Population structure and genetic diversity analyses

Population structure was estimated using the Bayesian model-based approach implemented in the software STRUCTURE v2.3.4 [23] and by Principal Component Analysis (PCA) in TASSEL v.5 [24] using SNPs with a minor allele frequency (MAF) >0.05. To identify the most likely number of subpopulations, STRUCTURE was run for each hypothetical number of subpopulations (*K*) between 1 and 8 using a burn-in period of 5000 iterations and a run length of 5000 Monte Carlo Markov Chain (MCMC) iterations. LnP(D) and Δ*K* values [25] were plotted with Structure Harvester [26]. After estimating the best *K*, a new run using a burn-in period of 100,000 and 100,000 MCMC was performed to assign accessions to subpopulations. Those accessions with a membership probability lower than 0.70 of belonging to one subpopulation were assigned to an ‘admixed’ group.

Principal Component Analysis (PCA) was conducted in TASSEL v.5 [24] on the same dataset and plotted using TIBCO Spotfire® 6.5.0.

A neighbor-joining (NJ) tree was generated based on Manhattan distances using the R package “Phyclust” [27].

Expected heterozygosity (*He*) and polymorphism information content (PIC) [28] were calculated for all *V. unguiculata* ssp. *unguiculat*a accessions and then separately for Iberian Peninsula accessions and for the worldwide set of accessions as in Muñoz-Amatriaín et al. [19].

SNP data were used to generate a similarity matrix between *V. unguiculata* ssp. *unguiculat*a accessions from Iberian Peninsula based on simple matching coefficient (number of common SNP alleles divided by the total number of SNPs).

## Results

### SNP genotyping and data curation

A high-density genotyping array containing 51,128 SNPs [19] was used to genetically characterize 43 landraces and cultivars from the Iberian Peninsula and 53 landraces collected worldwide for a total of 96 cowpea accessions. After SNP calling using GenomeStudio software (Illumina Inc., San Diego, CA, USA), quality control (QC) filtering was applied to both SNPs and accessions with the goal of removing SNPs with low performance accuracy, and accessions that failed in the SNP assay and/or were highly heterozygous (see Methods). Five accessions were eliminated, one of them (Ac61) because of its high percentage of missing calls (40%) indicating poor DNA quality, and the remaining four (Ac45, Ac46, Ac65 and Ac79) because they had high levels of “heterozygosity” (because DNAs were mixed from three plants, the apparent heterozygosity may have an alternative explanation of high heterogeneity between individuals), ranging from 22% to 33% heterozygous calls. These percentages exceeded the expected genetic variability within a cowpea landrace, where outcrossing rates from <1% to a maximum of 15% have been reported [3, 21, 22]. The remaining 91 accessions had percentages of heterozygosity from 0 to 16%, with an average of 2.7% heterozygosity.

A total of 44,056 good-quality polymorphic SNPs and 91 samples were used for further analysis. Pairwise SNP comparisons among accessions showed that Ac39 and Ac43 were potentially duplicates (100% similar SNP calls). These two accessions are members of ssp. *sesquipedalis* that were obtained from the National Plant Genetic Resources Centre-National Institute for Agricultural and Food Technology Research (CRF-INIA, Spain) genebank. This identity was also apparent at the phenotypic level (e.g. samples had the same growth habit, leaf type, flower color, seed color and shape, and hilum color).

### Genetic diversity and structure in the whole population

Genetic structure in the entire population of 91 accessions was evaluated using STRUCTURE v.2.3.5 [25], principal component analysis (PCA) in TASSEL V.5.0 [24] and a Neighbor-Joining (NJ) tree generated with “Phyclust” [27].

**Fig. 1.**
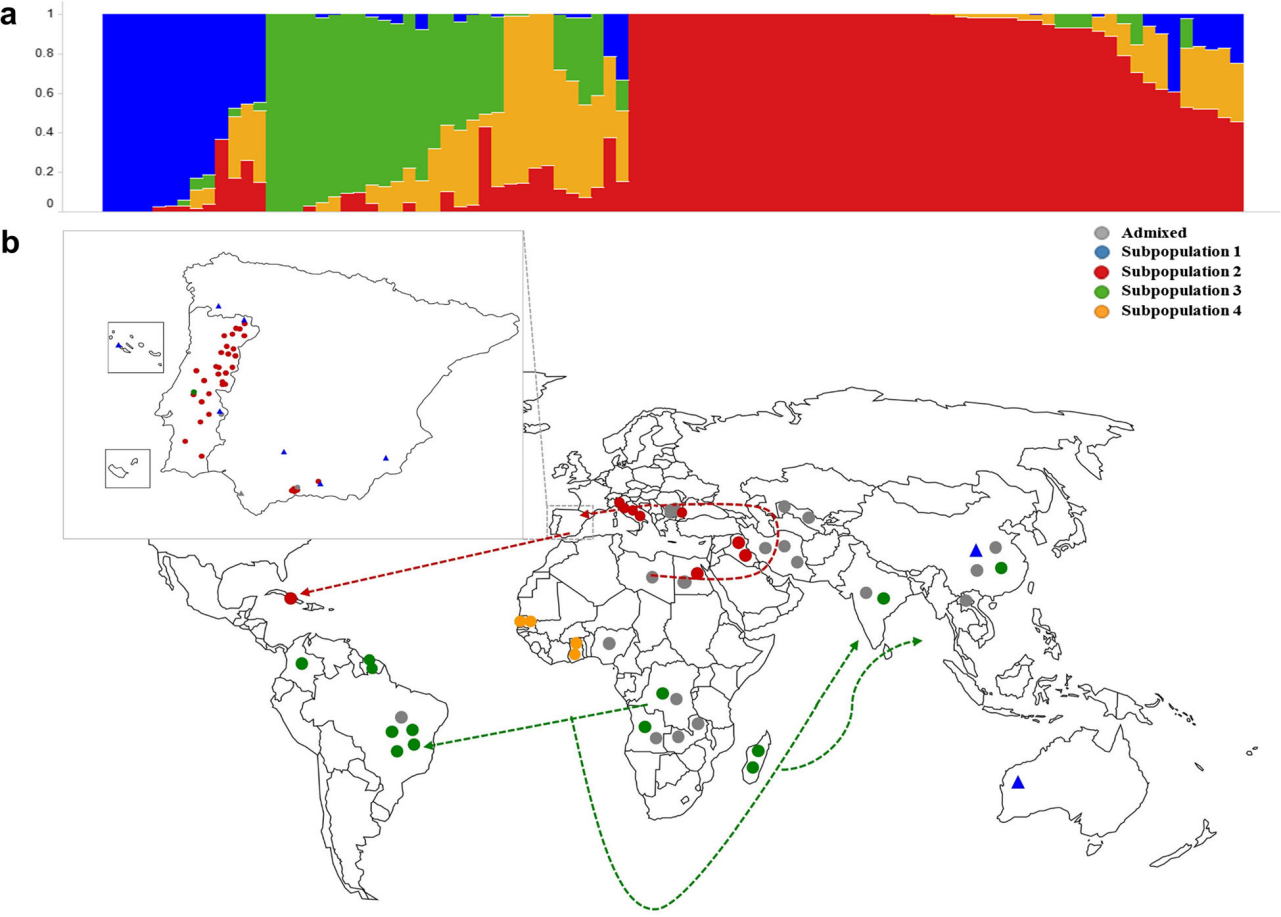
Population structure for 91 cowpea accessions. **a** Plot of ancestry estimates for K = 4; **b** geographical distribution and population structure of accessions used in this study, and inferred cowpea dispersion routes. Exact locations are provided for Iberian Peninsula accessions. For genebank accessions, coordinates were slightly adjusted in cases where latitude and longitude were identical to allow a visualization of all samples in the study. Each color represents a subpopulation as inferred by STRUCTURE (blue = subpopulation 1; red = subpopulation 2; green = subpopulation 3; orange = subpopulation 4), with ‘grey’ being used for the ‘admixed’ group (membership coefficient < 0.7). Shapes are used to distinguish the two subspecies of *Vigna unguiculata* used in this study, with circles representing ssp. *unguiculata* accessions and triangles indicating ssp. *sesquipedalis* accessions

Using STRUCTURE, the estimated log probability of the data for each given population (*K*), from 1 to 8, reached a maximum at *K* = 4 (Additional files 2 and 3). In addition, Evanno’s Δ*K* also showed the highest value at *K* = 4 (Additional files 2 and 3). These results indicated that the most likely number of subpopulations in this dataset is four. A new run was performed at *K* = 4 to assign accessions to subpopulations. Accessions with membership probability lower than 0.70 of belonging to one subpopulation were assigned to an ‘admixed’ group (Additional file 4). Subpopulation 1 included nine accessions, all of them members of ssp. *sesquipedalis*. All other subpopulations (2, 3, and 4) consisted of ssp. *unguiculata* accessions (Fig. 1; Additional file 4). Subpopulation 2 (41 accessions) included accessions from southern Europe, North Africa and Cuba; subpopulation 3 (13 accessions) included accessions from countries in South and Southeast Africa, South America and Asia; and subpopulation 4 (4 accessions) was composed of only West African accessions (Fig. 1; Additional file 4). The remaining 24 accessions were ‘admixed’.

**Fig. 2:**
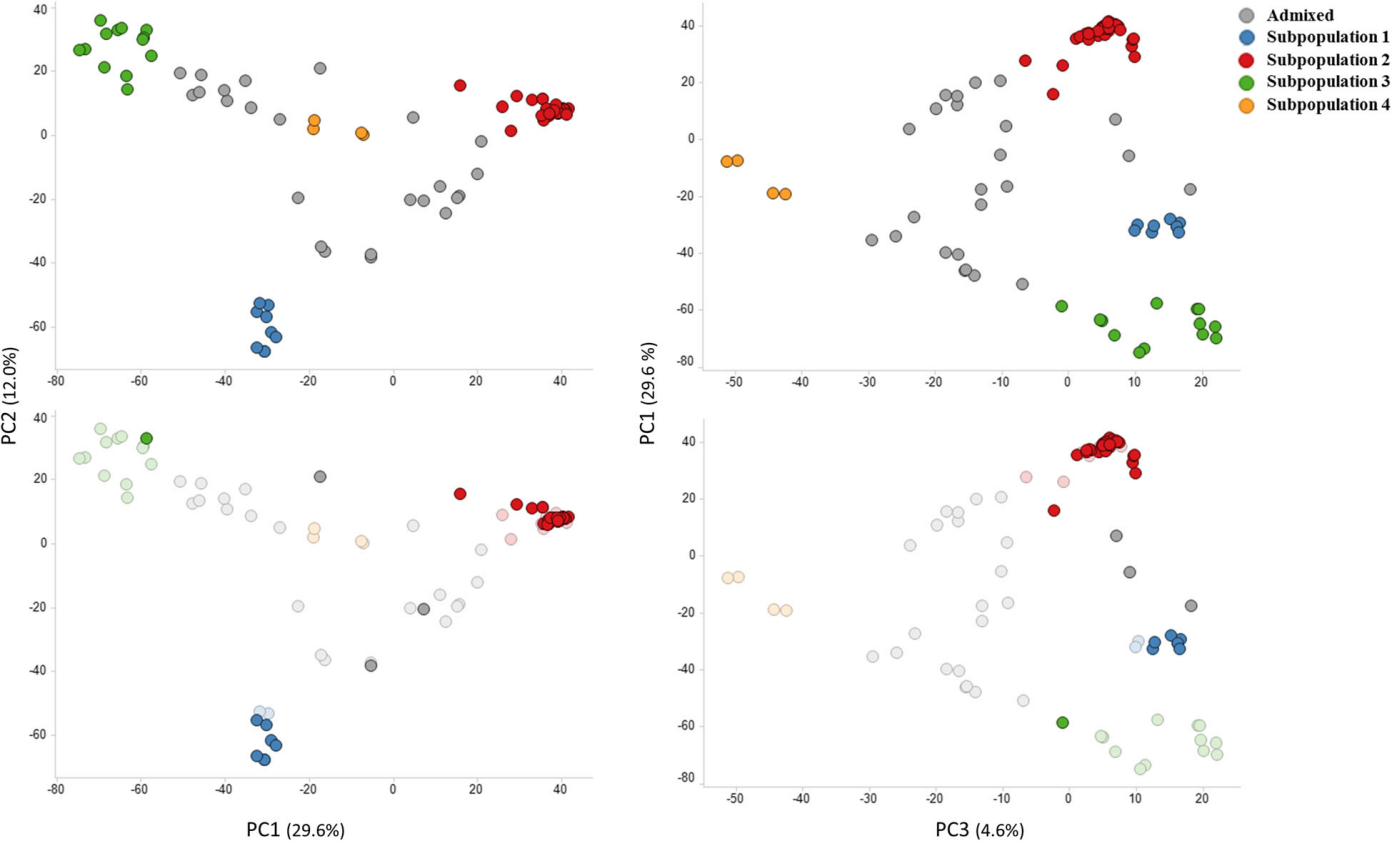
Principal component analysis of cowpea accessions used in this study. The accessions are colored by subpopulation membership (*K* = 4). Upper plots display all accessions, while the lower plots highlight only cowpea accessions from Iberia Peninsula

**Fig. 3:**
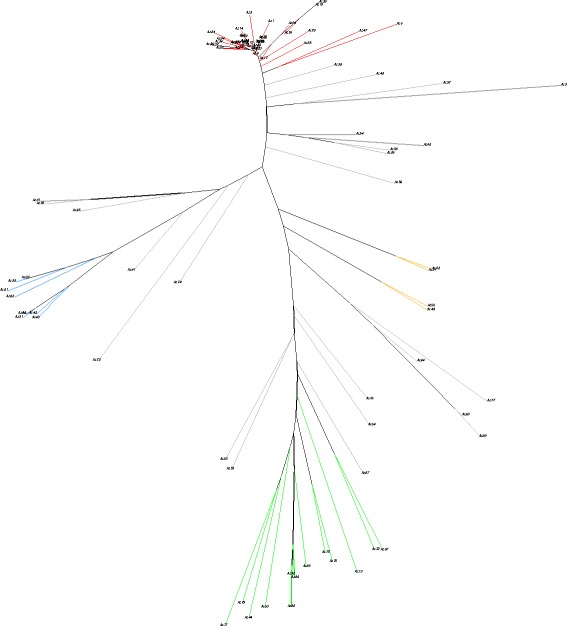
Neighbor-joining tree of 91 cowpea accessions with colors representing subpopulation membership (blue = subpopulation 1; red = subpopulation 2; green = subpopulation 3; orange = subpopulation 4; and grey = admixed)

This four major subpopulations were also distinguished by PCA (Fig. 2, upper plots): PC1 clearly separated subpopulations 2 and 3, while PC2 separated ssp. *sesquipedalis* accessions belonging to subpopulation 1 from the ssp. *unguiculata* ones. Subpopulation 4 was separated from the rest in PC3 (Fig. 2, upper plots). The NJ tree showed accessions clustered by subpopulation membership, supporting results from both STRUCTURE and PCA (Fig. 3).

PIC and *He* were calculated for the entire population and separately for each subpopulation (Table 1). Considering the whole dataset, the average PIC and *He* were 0.22 and 0.26, respectively. Average PIC values ranged from 0.07 in subpopulation 2 to 0.18 in subpopulation 3, while average *He* ranged from 0.09 to 0.23 in subpopulations 2 and 3, respectively (Table 1). This indicates that subpopulation 3 is the most diverse genetically, while subpopulation 2 appeared the least diverse, even though it contained the highest number of accessions (Table 1).

**Table 1:** Polymorphism information content (PIC) and expected heterozygosity (*He*) calculated for the entire population and for each subpopulation

Data set	N° accessions	N° countries	PIC	*He*
All accessions	91	24	0.22	0.26
Subpopulation 1	9	4	0.12	0.14
Subpopulation 2	41	7	0.07	0.09
Subpopulation 3	12	8	0.18	0.23
Subpopulation 4	4	2	0.12	0.15

The geographical distribution of accessions together with their subpopulation membership allowed inference of possible dispersion routes (Fig. 1). The similarity between European and northern African accessions seems to indicate that cowpeas were brought by Arabs to Europe. The accession from Cuba may have been brought by Spanish navigators because Cuba was a Spanish colony and consequently commercial exchanges were frequent. The accessions from South America and Asia belonged to the same subpopulation as those from South/East Africa (Fig. 1). It is possible that these were brought from that region in Africa to Asia and South America during the discovery period, when Portuguese had an important role in commercial routes in the southern hemisphere. If so, Iberian Peninsula people may have had an important role in the distribution of cowpea from Africa and Europe to other parts of the world.

### Genetic structure and diversity of Iberian peninsula accessions from subspecies *unguiculata*

Genetic structure and diversity were explored for 35 Iberian Peninsula accessions belonging to ssp. *unguiculata* compared to 46 world-wide ssp. *unguiculata* accessions. Due to the low number of ssp. *sesquipedalis* accessions in the dataset (10 in total) and the fact that grain-type cowpea (ssp. *unguiculata*) is the most cultivated and consumed in Europe, ssp. *sesquipedalis* accessions were not included in these analyses. Most of the 35 *V. unguiculata* ssp. *unguiculata* accessions from the Iberian Peninsula belonged to subpopulation 2, together with other Genebank accessions from Europe (Fig. 2, lower plots; Additional file 4). Only two accessions from Portugal (Ac5 and Ac13) and one accession from Spain (Ac38) did not belong to this subpopulation: Ac13 belonged to subpopulation 3, while accessions Ac5 and Ac38 were considered admixed (estimated proportion of subpopulation 2 = 0.43 and 0.61, respectively). These three accessions would then likely contain unique alleles not present in any other Iberian Peninsula accession studied. An examination of the SNP data from all 35 Iberian Peninsula accessions showed that, of all polymorphic SNPs (29,550) in the Iberian Peninsula dataset, 4777 were contributed only by Ac13 (16.2%). These unique alleles from Ac13 were distributed all over the linkage groups (LGs; Additional file 5). As expected, Ac5 and Ac38 contained a lower number of unique alleles, 1849 (6.3%) for Ac5 and 534 (1.8%) for Ac38. Unique alleles from Ac5 were found in all cowpea chromosomes, while those from Ac38 were mainly present on the pericentromeric region of LG3 and LG11, and towards the distal end of LG8 (Additional file 5).

PIC and *He* were calculated for the entire set of 81 *V. unguiculata* ssp. *unguiculata* accessions, and then separately for Iberian Peninsula accessions and for those from other countries (Table 2). Considering the ssp. *unguiculata* whole dataset, average PIC and *He* were 0.21 and 0.25, respectively. PIC and *He* values were quite different between accessions from the Iberian Peninsula (0.09 and 0.10, respectively) and those from the world-wide collection (0.25 and 0.31, respectively). This indicates that genetic diversity in Iberian Peninsula ssp. *unguiculata* accessions is low compared to the diversity available in the world-wide sample of cultivated cowpeas. To better understand and compare accessions from the Iberian Peninsula at the genetic level, similarity matrix was generated based on comparisons between all 35 accessions (Additional file 6). From this it was apparent that Ac13, Ac5 and Ac38 had the lowest similarity indexes with the rest of the Iberian Peninsula accessions. This was expected since they had the lowest genomic ancestry proportions of subpopulation 2, to which all other Iberian Peninsula accessions belong (Additional file 4). The other 32 accessions were very similar to each other, with percentages of similarity ranging from 77.0% to 99.9%.

**Table 2:** Polymorphism information content (PIC) and expected heterozygosity (*He*) calculated for *V. unguiculata* ssp. *unguiculata* accessions

Data set	N° accessions	N° countries	PIC	*He*
All *V. unguiculata* ssp. *unguiculata* accessions	81	23	0.21	0.25
Iberian Peninsula accessions	35	2	0.09	0.10
Accessions from other countries	46	21	0.25	0.31

## Discussion

Genetic characterization of germplasm resources is essential for conservation and the sustainable use of their diversity [29]. In recent years, several studies have characterized cowpea germplasm mainly from Africa and Asia [13, 30–33]. However, there have been no studies exploring in depth the genetic diversity of southern European cowpeas.

In this study, high-density SNP genotyping using the Cowpea iSelect Consortium Array [19] has provided a means to study population structure and genetic diversity in a set of 91 world-wide cowpea accessions, with a special focus on 43 accessions from the Iberian Peninsula. A high proportion of the SNPs assayed by the array were polymorphic in the dataset (44,056 of 51,128; 86%). Also PIC and *He* values obtained from the entire population are similar to those reported by Huynh et al. [34] and Muñoz-Amatriaín et al. [19] using a larger dataset, indicating that the selection of worldwide accessions in the present work provides a good representation of the diversity in cultivated cowpea.

The SNP genotyping of these accessions enabled identification of one apparent duplication: Ac39 and Ac43, which are members of the subspecies *sesquipedalis*. These were provided by the National Plant Genetic Resources Centre-National Institute for Agricultural and Food Technology Research (CRF-INIA, Spain) genebank, and their passport information is limited. Ac39 and Ac43 are both from Spain, but from two different regions: Ac1 is from Cordoba (Andalucia region, south of Spain) and Ac43 from Ourense (Galicia region, north of Spain). A common cause of redundant accessions is the unwitting submission of the same accession to the genebank, then generating more than one name or designator. Identifying these redundant accessions is not possible using phenotype data alone [35]. Duplicated accessions do not contribute to genetic diversity of collections while generating unnecessary and additional costs to genebank [36].

The population structure analysis assigned the 91 accession to four subpopulations. In agreement with the results of Huynh et al. [34] and Xiong et al. [37], two of the subpopulations identified (subpopulation 3 and subpopulation 4) corresponded to the East/South Africa and the West Africa gene pools, respectively. In addition to those two genetic clusters, our study identified two more subpopulations composed of North Africa and South Europe accessions (subpopulation 2) and *V. unguiculata* ssp. *sesquipedalis* accessions (subpopulation 1). The aforementioned studies may not have identified those two populations because of a lack of accessions from these regions.

The geographic distribution of the accessions from the three ssp. *unguiculata* subpopulations enabled inference of possible dispersion routes of domesticated cowpea (Fig. 1). It has been reported that some Iberian Peninsula crops were introduced in Europe through the “Arab corridor” [38]. Our study is consistent with the idea that cowpea was one of the crops brought by Arabs from North Africa to Europe in ancient times. From the end of the fifteenth century until the middle of the seventeenth century, Portugal and Spain, which form the Iberian Peninsula, had an important role in the great discovery period. Saúco and Cubero [38] described how powers from the Iberian Peninsula had an important contribution to the exchange and acclimatization of new and old world crops, including cowpea, due to exploration voyages and commercial routes established by them. This information together with the genetic data from this study seems to indicate that the accession from Cuba (Ac62) belonging to subpopulation 2 may have been brought by the Spaniards. This island was discovered in 1492 by Christopher Columbus and belonged to Spain until 1898, so it seems plausible that the Spaniards introduced this crop to Cuba. On the other hand, Portuguese sailors explored and dominated the Southern hemisphere including South America (more specifically Brazil), Southern Africa (Angola, Guinea Bissau, Mozambique) and India. They established direct contact between Europe, South America and India, and later with Southeast Asia and China [38]. Since subpopulation 3 includes accessions from all these regions, it is possible that slaves being transported in Portuguese ships crossing the Atlantic Ocean were the ones who introduced cowpea cultivation into Brazil. Additional cowpea introduction into India and later China may also have occurred through the Portuguese sea routes as well.

Cowpea genetic diversity among countries and regions can be affected by environmental factors and customs of cowpea consumption [37]. In the Iberian Peninsula, cowpea is a minor crop, mostly based on cultivation of landraces. These landraces reflect the cultural identity of local people and are reservoirs of diversity for breeding improvement. Given the narrow genetic base found in this study for most of the Iberian Peninsula cowpea, introduction of additional diversity into the Iberian Peninsula genepool seems sensible to keep increasing yields under changing climatic conditions [29]. Three of the accessions belonging to the Iberian Peninsula were more diverse than the rest: Ac13 was the most different from the others and had mostly subpopulation 3 ancestry, while Ac5 and Ac38 had admixed ancestry from subpopulations 2 and 3, and subpopulations 1 and 2, respectively (Additional file 4). Ac5 is a variety developed by breeders at INIAV-Elvas (Portugal) and Ac38 is a landrace from Spain. Given its proportion of ancestry from subpopulation 3 (0.50), Ac5 may have resulted from crosses between accessions from the Iberian Peninsula and South/East African materials. Although Ac38 is morphologically similar to other ssp. *unguiculata* accessions, its genome has an estimated proportion of subpopulation 1 ancestry of 0.39 (Additional file 4). This accession could be the result of intentional crosses between the two cultivar-groups. The introduction of Ac13, a member of subpopulation 3, into Portugal could have occurred in the 70’s. During that time, Portuguese living in Angola, Guinea and Mozambique returned to Portugal and could have brought that cowpea landrace with them. It is also possible that during the great discover period navigators brought that accession from Africa, Asia or South America (Brazil). The aforementioned accessions Ac5, Ac13 and Ac38 can be very useful for breeding programs as they can bring additional genetic diversity without compromising adaptation to the environment.

## Conclusions

Higher cowpea production is needed in Europe to meet demand, and only Southern European countries possess climatic conditions that are favorable for growing this legume crop. Here we have genetically characterized a geographically diverse set of cowpeas that are cultivated in the Iberian Peninsula using a high-density genotyping array, and we have compared them to cowpea accessions collected world-wide. Our study identified four subpopulations in the whole dataset, with most Iberian Peninsula accessions of ssp. *unguiculata* belonging to the same subpopulation and having lower levels of genetic diversity than world-wide cowpea accessions. However, we identified one Iberian Peninsula landrace with ancestry from another subpopulation and two accessions having admixture of different subpopulations. These three accessions may be used to incorporate new genetic diversity into breeding programs without compromising adaptation. Possible dispersion routes of cultivated cowpea have been also inferred using the SNP data combined with passport information. In the future, favorable alleles for simple and complex traits could be mined from these accessions via genome-wide association studies.

## Additional files

Additional file 1: Information on cowpea accessions used in this study. (XLSX 12 kb)

Additional file 2: Raw STRUCTURE output for all runs (left) and Δ*K* calculations for each number of ***K*** (right). (XLSX 12 kb)

Additional file 3: Exploration of the optimal number of subpopulations (*K*) in the entire dataset. Plots were generated with Structure Harvester [26]. (A) Estimated log probability of the data for each *K* between 1 and 8. (B) Δ*K* values as a function of *K*. (TIFF 75 kb)

Additional file 4: Genetic structure information on the 91 accessions. The estimated membership of each accession in the four subpopulations is shown, as well as the PCA coordinates. (XLSX 17 kb)

Additional file 5: Genomic location of unique alleles in Ac13, Ac5 and Ac38 on cowpea linkage groups (LGs). Genomic regions colored in red contain unique alleles in the corresponding accession, while regions containing non-unique alleles are represented in blue. For the figure, one marker per locus was kept, giving priority to unique alleles over non-unique ones. In white are represented regions lacking mapped SNPs. LG number and cM positions are based on the cowpea consensus genetic map available from Muñoz-Amatriaín et al. [19]. (TIFF 2302 kb)

Additional file 6: Matrix showing genetic pair-wise similarity values for Iberian Peninsula accessions. (XLSX 16 kb)

## Abbreviations

BC: Before Christ
CRF-INIA: National Plant Genetic Resources Centre-National Institute for Agricultural and Food Technology Research
CTAB: cetyl trimethyl ammonium bromide
DNA: Deoxyribonucleic acid
EMBRAPA: Brazilian Agricultural Research Corporation
GWAS: Genome-wide association studies
*He*: Expected heterozygosity
INIAV: National Institute for Agrarian and Veterinarian Research
IPK: Leibniz Institute of Plant Genetics and Crop Plant Research
LG: Linkage group
MAF: Minor allele frequency
MCMC: Monte Carlo Markov Chain
NJ: Neighbor-joining
PCA: Principal component analysis
PIC: Polymorphism Information Content
QC: Quality control
QTL: Quantitative Trait Locus
SNP: Single Nucleotide Polymorphism
