# Supplementary figures and images for: Genetic diversity and structure of Iberian Peninsula cowpeas compared to world-wide cowpea accessions using high density SNP markers
Source: BMC Genomics. 2017 Nov 21;18:891. doi: 10.1186/s12864-017-4295-0 (PMC5697113; doi:10.1186/s12864-017-4295-0)

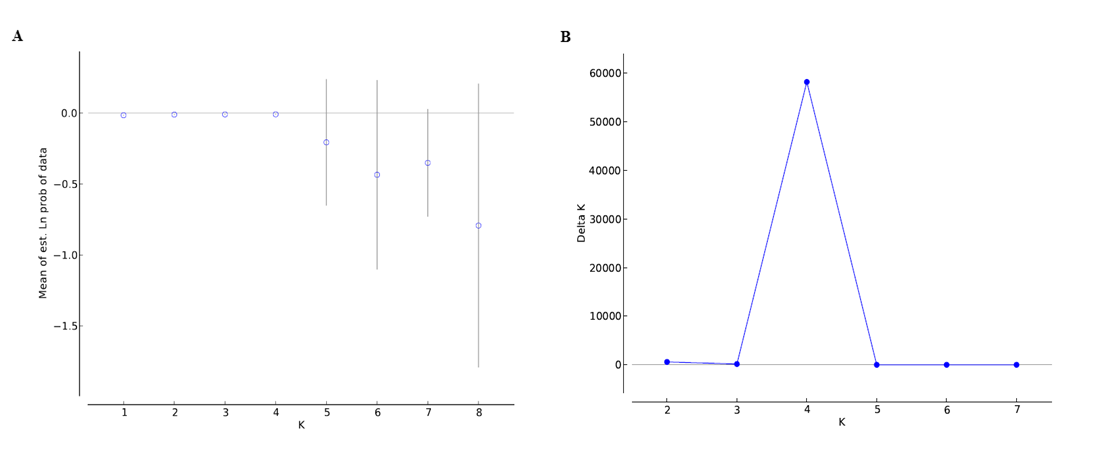

Supplement: Supplementary file 3 — Exploration of the optimal number of subpopulations (K) in the entire dataset. Plots were generated with Structure Harvester [26]. (A) Estimated log probability of the data for each K between 1 and 8. (B) ΔK values as a function of K. (TIFF 75 kb) [file 12864_2017_4295_MOESM3_ESM.tif]

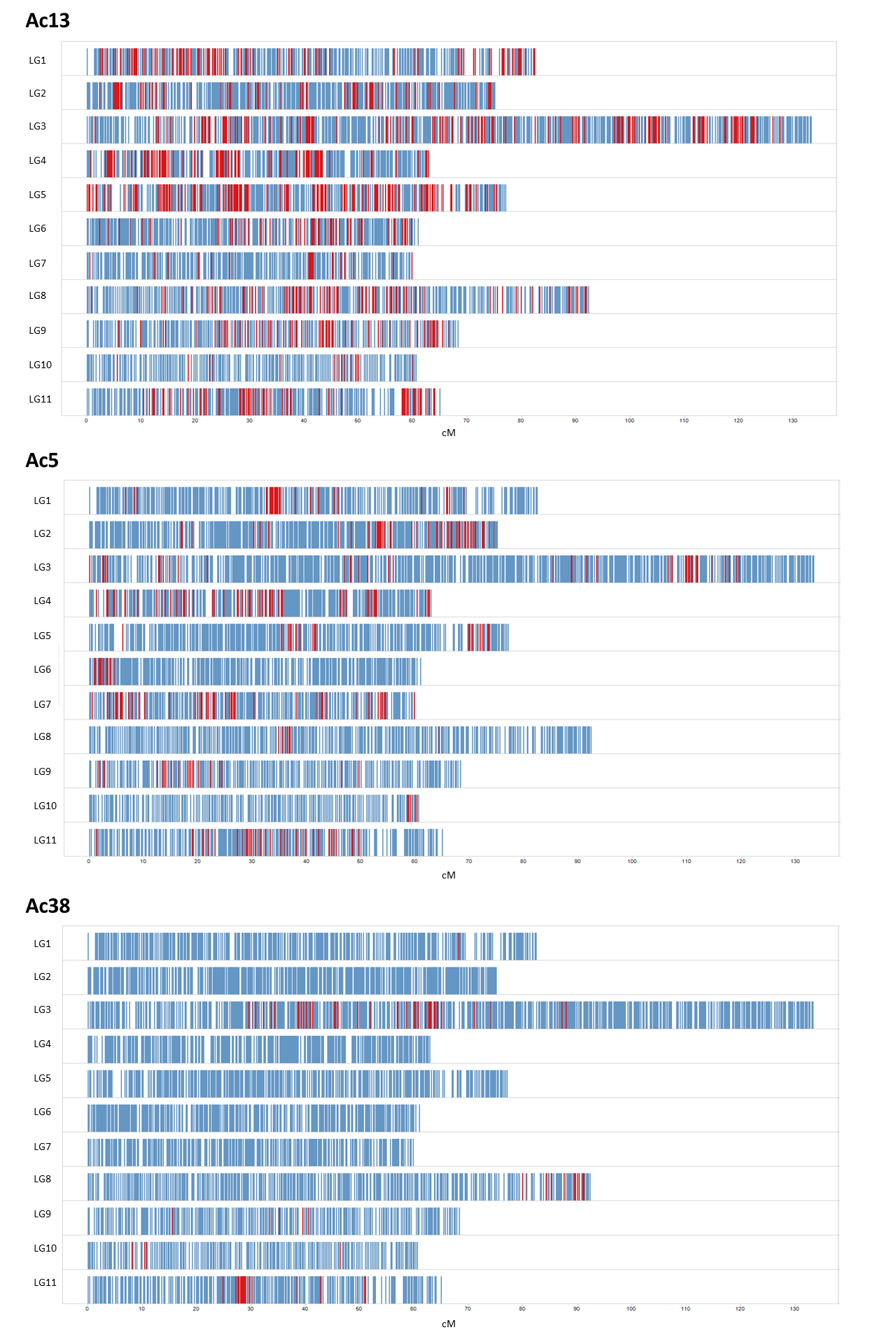

Supplement: Supplementary file 5 — Genomic location of unique alleles in Ac13, Ac5 and Ac38 on cowpea linkage groups (LGs). Genomic regions colored in red contain unique alleles in the corresponding accession, while regions containing non-unique alleles are represented in blue. For the figure, one marker per locus was kept, giving priority to unique alleles over non-unique ones. In white are represented regions lacking mapped SNPs. LG number and cM positions are based on the cowpea consensus genetic map available from Muñoz-Amatriaín et al. [19]. (TIFF 2302 kb) [file 12864_2017_4295_MOESM5_ESM.tif]
